# Essential oils as nature-engineered antifungals: a comprehensive review of pharmacological mechanisms, drug synergism, and advanced formulation strategies

**DOI:** 10.3389/fphar.2026.1799031

**Published:** 2026-04-17

**Authors:** Harshini Jalendiran, Aditi Roy, Sahasrakshi Sathiyanarayanan, Swethavarshini Geethanarayanan, Abarna Geethanjali Kannan Selvam, Harshavarthini Vijayasekaran, Sudha Ramaiah, Anand Anbarasu

**Affiliations:** 1 Department of Biotechnology, School of Biosciences and Technology, Vellore Institute of Technology (VIT), Vellore, Tamil Nadu, India; 2 Medical and Biological Computing Laboratory, School of Biosciences and Technology (SBST), Vellore Institute of Technology (VIT), Vellore, Tamil Nadu, India; 3 Department of Biosciences, School of Biosciences and Technology, Vellore Institute of Technology (VIT), Vellore, Tamil Nadu, India

**Keywords:** antifungal activity, EOS, ergosterol inhibition, phenolic terpenoids, synergy

## Abstract

Essential Oils (EOs) and their active compounds are more effective than traditional antifungal drugs. They are effective against a wide range of pathogens and can neutralise multiple targets simultaneously. Fungal resistance development is minimised due to multi-target effects. EOs that are rich in phenols and terpenoids are derived from plants such as *Cinnamomum*, *Thymus*, *Origanum*, and *Syzygium*. These compounds act against fungal pathogens, namely, *Candida*, *Aspergillus*, and dermatophyte fungi. They function by disrupting cell membranes and inhibiting ergosterol biosynthesis in fungi. Oxidative stress also plays a significant role in this context. When antifungal agents such as amphotericin B and fluconazole are combined with active compounds in EOs, they create synergy, increasing the efficacy of the active compounds while reducing the drug dose. This decreases the toxicity of the drugs. In recent days, new drug manufacturing processes, such as nano emulsions and natural deep eutectic solvents, have emerged, thereby increasing the stability of volatile compounds in EOs. This increases the drug’s solubility, thereby enhancing its absorption. This review highlights the mechanisms by which EOs act against fungi, the mechanisms involved, and their compatibility with other treatments.

## 1 Introduction

The global burden is the prevalence of invasive fungal infections that cause 1.7 million deaths annually. *Candida* species are responsible for many of these infections (Lyman et al., 2023). They are the primary cause of systemic fungal infections and rank among the top causes of bloodstream infections in hospitals. Invasive candidiasis has high mortality rates (∼40% in severe cases) (Lyman et al., 2023; Esmaeili et al., 2025). These infections range from conditions such as oral, vaginal, and skin infections to severe, life-threatening bloodstream infections (Konuk and Ergüden, 2020; Thompson and Young, 2021; Hou and Huang, 2024). With the rise of non-*albicans* species and emerging multidrug - resistant pathogens like *Candida auris,* the clinical spectrum has become more difficult to manage (Köhler et al., 2017; Thompson and Young, 2021; Noble et al., 2023).

Conventional antifungal agents rely on a few drug classes (azoles, polyenes, echinocandins, pyrimidines) (Pérez-Cantero et al., 2020). However, the resistant strains decrease the effectiveness of these drugs, particularly against azoles, which significantly reduced therapeutic options in recent years (Konuk and Ergüden, 2020; Geremia et al., 2023; Esmaeili et al., 2025). Meanwhile, the prolonged use of drugs like amphotericin B and azoles is limited since they lead to nephrotoxicity and hepatotoxicity, respectively (Noble et al., 2023). There are many promising compounds that show *in vitro* activity but fail to translate into clinical efficacy due to bioavailability and stability issues, which is considered a translational gap (Esmaeili et al., 2025).

EOs are plant - derived extracts with their biological activities, widely used in traditional medicine and food preservation due to their antimicrobial and preservative properties (Sharifi-Rad et al., 2017; Ghosh et al., 2025a; Mazumder et al., 2026). They have gained scientific interest as potential antifungal agents, focusing on their chemical composition, modes of action, and clinical efficacy against relevant fungi (Singh, 2001; Kong et al., 2019; Martins and Bicas, 2024). These EOs, with their multi-target mechanisms of action, reduce the likelihood of resistance. Their synergistic potential with existing antifungal agents such as fluconazole and amphotericin B enhances efficacy, lowers drug doses, and helps overcome drug resistance in fungal strains (Konuk and Ergüden, 2020; Bravo-Chaucanés et al., 2022; Gupta and Venkataraman, 2022; Hou and Huang, 2024).

Antifungal actions of EOs are mediated by various cellular and molecular mechanisms. Their lipophilicity enables them to become part of fungal cell membranes and make them fluid and permeable that results in the leakage of ions and intracellular components and eventual cell death (Casadevall et al., 2019; Konuk and Ergüden, 2020). Some of them also block the production of ergosterol, which interferes with membrane integrity and function, and others disrupt important enzymes involved in cell wall production. Moreover, EOs may disrupt the functioning of mitochondria and cause overproduction of reactive oxygen species (ROS), which cause oxidative stress and apoptosis (de Sousa et al., 2023). Terpenoid phenols also increase antifungal effects by disrupting calcium and interrupting nutrient-sensing signalling pathways, including TOR, and sensitizing fungi to stress and antifungal agents (Rao et al., 2010). Due to its complicated chemical structure and synergistic activity, EOs have the potential to target various cellular targets, which reduces the risk of developing resistance (Basu et al., 2023). They have a high level of antifungal activity against *Candida albicans*, *C. auris*, dermatophytes, and *Aspergillus,* they frequently outperform conventional drugs *in vitro* (D’Auria et al., 2005; Kedia et al., 2014). They have been shown to exhibit multi-target activity, thereby reducing resistance to single-target antifungals. They are also synergistic with fluconazole and amphotericin B, including resistant *C. auris* (Bravo-Chaucanés et al., 2022; Cavallo et al., 2024). Besides, EOs cause disruption of biofilms and are less toxic in optimized preparations, which contribute to their application as safer complementary treatments (Kedia et al., 2014).

Recent computational approaches have significantly accelerated the identification of bioactive phytochemicals and their therapeutic potential through molecular modeling and systems biology approaches (Miryala et al., 2022; Ghosh et al., 2025b; Joshi et al., 2025). The current review aims to provide an overall examination of the antifungal potential of EOs, explaining the pathways by which they work and their capacity to circumvent traditional resistance mechanisms (Konuk and Ergüden, 2020). In addition, formulation and delivery strategies, such as Natural Deep Eutectic Solvents (NADES) and nano formulations, which improve stability and therapeutic use (Espino et al., 2019; Merad et al., 2021; Pezantes-Orellana et al., 2024). This review further identifies the research gaps, which include a lack of clinical trials, issues of standardization, and limited safety data, which point to future investigations (Esmaeili et al., 2025).

## 2 Source and composition of EOs

Aromatic and medicinal plants of families, namely, *Lamiaceae, Myrtaceae*, and *Lauraceae* give the strongest antifungal EOs (Khoury et al., 2016). The most well-researched ones are cinnamon (*Cinnamomum verum*), thyme (*Thymus vulgaris*), oregano (*Origanum vulgare*), clove (*Syzygium aromaticum*), and tea tree (*Melaleuca alternifolia*), whose oils are a source of phenolic and terpenoid compounds that predetermine their bioactivity (Khaleel et al., 2018; Aimad et al., 2022; Souza et al., 2024). Despite the significant role of thymol, carvacrol, and eugenol as the main antifungal components that cause the formation of membrane disruption and intracellular leakage, the antifungal activity cannot be attributed to major compounds alone. The less active minor constituents, such as p-cymene and γ-terpinene, increase membrane fluidity and permeability, thereby promoting improved permeation and accumulation of phenolic compounds in cells. This suggests that the antifungal activities of EOs are mainly due to synergistic effects of the whole oil, as opposed to the effect of the major constituents (Gao et al., 2016; Kong et al., 2019). The antifungal activity of whole essential oils (EOs) is due to the combined action of both major and minor components. Therefore, the bioactivity results from interactions among different compounds rather than from a single dominant component alone. *Lonicera japonica* and *Satureja montana* are other less-utilized sources that have also been tested with good antifungal properties and unique profile of metabolites (Rahman, 2014; Yuan et al., 2024). The variation of antifungal efficacies of the EOs can be attributed to compositional differences, which depend on species, environmental factors, age of plant and extraction procedures adopted as listed in Table 1 (Hudaib et al., 2002; Fahed et al., 2017).

**TABLE 1:** Source and composition of EOs.

Botanical source	Major components	Extraction method	Antifungal target	References
*Cinnamomum verum* (Cinnamon)	Cinnamaldehyde, Eugenol	Steam distillation	*Candida albicans*, *Candida auris*, *Aspergillus* spp.	Yuan et al. (2024)
*Thymus vulgaris* (Thyme)	Thymol, Carvacrol, Linalool	Steam distillation	*Penicillium digitatum*, *Candida albicans*	Diánez et al. (2018)
*Origanum vulgare* (Oregano)	Carvacrol, Thymol, p-Cymene	Steam distillation	*Candida* spp.*, Dermatophytes*	Daferera et al. (2003)
*Syzygium aromaticum* (Clove)	Eugenol, Caryophyllene	Steam distillation	*Penicillium digitatum*, *Candida* spp.	Souza et al. (2024)
*Melaleuca alternifolia* (Tea Tree)	Terpinen-4-ol, γ-terpinene, α-terpinene	Steam distillation	*Candida* spp.*, Dermatophytes*	Kong et al. (2019)
*Lonicera japonica*	Phenolics, Alkaloids (unspecified)	Solvent extraction	*Microsporum canis*, *Trichophyton* spp.	Jiménez-Reyes et al. (2019)
*Satureja montana*	Carvacrol, γ-terpinene	Steam distillation	*Candida* spp.	Diánez et al. (2018)
*Cymbopogon martinii* (Palmarosa)	Geraniol, Geranyl acetate	Steam distillation	*Candida* spp.	Falleh et al. (2020)
*Eucalyptus citriodora* (Lemon eucalyptus)	Citronellal, Citronellol	Steam distillation	*Penicillium* spp.*, Candida* spp.	Souza et al. (2024)
*Backhousia citriodora* (Honey myrtle)	Citral, Neral, Geraniol	Steam distillation	*Candida* spp.	Falleh et al. (2020)

### 2.1 Major and minor bioactive components

The antifungal effects of the main essential oil constituents, which are discussed in this review (thymol, carvacrol, terpinen-4-ol, and eugenol) are highly regulated by specific structure-activity relationships (SAR) depending on their functional group and physicochemical characteristics. Thymol and carvacrol are phenol monoterpenes that have been protonophoric owing to the existence of the hydroxyl (-OH) group that is attached to an aromatic ring and exhibits strong antifungal activity. Phenolic hydroxyl groups facilitate cross-membrane proton exchange between fungal cells, leading to membrane depolarization, increased permeability, ion efflux, and disruption of intracellular homeostasis. Their hydrophobic aromatic backbone helps them insert into ergosterol-rich lipid bilayers and destabilize the membranes. Cinnamaldehyde has an α, β-unsaturated aldehyde group, which confers electrophilic reactivity and thus interacts with thiol-containing fungal proteins and enzymes associated with membrane depolarization via Michael-type addition reactions, disrupting metabolism (Yuan et al., 2024). The monoterpene alcohol Terpinen-4-ol, with no aromatic ring, is a major perturbation of lipid bilayers and induces higher membrane fluidity, although due to the lack of the phenolic proton-donating group, it has less depolarizing potential than thymol and carvacrol. Eugenol is a phenolic compound containing an allylic side chain, which enables it to interact with and insert into cellular membranes, thereby inducing oxidative stress. The presence of the phenolic hydroxyl group allows the exchange of protons, whereas the allyl side chain increases lipophilicity and membrane affinity. Together, antifungal efficacy is related to the availability of proton-donating phenolic groups, electrophilic carbonyl functional groups, and structural components that facilitate membrane partitioning, which offers a mechanistic explanation for the varying efficacy of these EO constituents (Gao et al., 2016; Khaleel et al., 2018). As a result, the quantitative and qualitative characteristics of EOs determine their biological activity and particularity, and each study of antifungal activity requires uncompromising chemical profiling (Kong et al., 2019).

### 2.2 Methods for extraction and characterization

#### 2.2.1 Extraction methods

The extraction procedure has a profound effect on the yield and chemical composition of EOs, as well as on their reported antifungal activity. Additionally, steam distillation has consistently been found to yield oils rich in volatile phenolic acids, such as thymol, carvacrol, and eugenol, which are strongly associated with low minimum inhibitory concentration (MIC) values and superior antifungal activity; thus, in comparative studies, steam-distilled oils tend to show greater antifungal activity. The high temperatures involved, however can induce degradation of thermolabile constituents, causing loss in the activities of some plants (Hudaib et al., 2002). By comparison, solvent extraction with ethanol or methanol will extract more oxygenated compounds and less volatile compounds, which can be advantageous in certain systems, but may also cause compositional variability and solvent residue issues, restricting pharmaceutical or food use (Rahman, 2014). NADES have been more recently demonstrated to preserve heat-sensitive compounds and preserve synergistic activity of constituents, and several studies have shown that extraction methods that maintain chemical integrity and synergy of entire extracts can also greatly change the antifungal activity (Espino et al., 2019). Pharmaceutical and topical formulations are promising for use with these novel systems.

Notably, there is some comparative evidence that the extraction methodology can substantially affect the reported antifungal results in studies. When phenolic monoterpenes such as thymol, carvacrol, and eugenol serve as the principal antifungal constituents, steam-distilled essential oils often exhibit lower minimum inhibitory concentration (MIC) values than solvent extracts. This occurs because these volatile compounds are preferentially concentrated in the distillate during steam distillation. Conversely, the solvent-based extracts can reduce the phenolic strength by co-extracting non-volatile lipids, waxes and polar secondary metabolites that may change the observed antifungal profile. Nevertheless, when working with plant matrices containing large quantities of thermolabile aldehydes, such as cinnamaldehyde, long hydrodistillation times can reduce bioactive recovery, whereas low-intensity supercritical CO2 extraction conditions can preserve oxygenated and heat-sensitive components, commonly leading to an increase in antifungal activity. Equally, microwave-assisted extraction (MAE) enhances cell wall disruption and reduces extraction time, thereby improving the recovery of active monoterpenes, but compositional imbalance can occur without optimizing power and extraction time. These findings suggest that differences in MIC values across studies may, in part, be due to extraction-induced compositional changes rather than inherent differences in antifungal activity.

#### 2.2.2 Chemical characterization techniques

Volatile properties of EOs are most often analysed with the help of Gas Chromatography-Mass Spectrometry (GC-MS) (Ahmad Khan et al., 2019; Hou and Huang, 2024). Using GC-MS enables the identification of essential terpenes, aldehydes, and phenolic compounds, and the correlation of chemical composition with antifungal activity. Nevertheless, essential oil profiling is not an aspect of inter-laboratory verification because there is no standardization of critical analytical criteria. In most experiments, mass spectral library matching is used to identify compounds without routine confirmation using retention indices (RI), whereas in others, various RI databases are used, or none are used at all. Also, because relative peak areas are used rather than absolute concentrations, quantification is often not carried out, preventing a definitive comparison of the abundance of any compound and its antifungal activity across laboratories and analytical platforms.

Non-volatile or thermolabile compounds are better analysed by high-performance liquid chromatography (HPLC) and liquid chromatography-mass spectrometry (LC-MS), which are better at both separation and quantification; however, differences in ionization methods, calibration standards, and data processing can also introduce variability. Such methodological discrepancies point to the necessity of consistent methods of analysis in the process of ensuring the credibility of the necessary comparisons of the essential oil composition and the ability to correctly correlate the chemical profiles with the antifungal activity (Espino et al., 2019; Pezantes-Orellana et al., 2024).

Despite the extensive use of GC-MS for essential oil characterization, there is substantial variation in instrumentation, column choices, and operating conditions across studies. Table 2 summarizes the most frequently reported GC-MS types in antifungal essential oil studies. This variability has demonstrated the need for standardized reporting of analyses to compare chemical profiles and assess reproducibility.

**TABLE 2:** GC-MS analytical techniques that are typically applied in antifungal research on the essential oil profiling.

Type of GC-MS	Role in antifungal EO studies	References
Single quadrupole GC–MS (Electron Ionization mode)	Most widely used configuration for routine qualitative identification of EO constituents through electron ionization spectra and library matching	Hudaib et al. (2002)
GC–MS/MS (Triple quadrupole)	Enables targeted analysis and confirmation of specific EO constituents with improved selectivity and sensitivity	Marriott et al. (2001)
GC–TOF–MS (Time-of-Flight)	Provides high-resolution detection and accurate mass measurement for complex terpene mixtures	Marriott et al. (2001)
GC–QTOF–MS (Quadrupole time-of-flight)	Allows accurate mass identification and improved characterization of minor EO components	Marriott et al. (2001)
Ion-trap GC–MS	Allows MS^n^ fragmentation analysis for structural elucidation of terpenoids and phenolic EO compounds	Marriott et al. (2001)

## 3 Antifungal spectrum of EOs

### 3.1 *In vitro* activity against *Candida* species

Various EOs and active products derived from these oils have shown significant antifungal activity *in vitro* against *C. albicans* and non-*albicans Candida* species. In this review, the reported MIC values are derived from studies evaluating essential oils and their major constituents in different experimental forms. Thymol and carvacrol are primarily discussed as dominant phenolic constituents of crude essential oils such as thyme (*T. vulgaris*) and oregano (*O. vulgare*) oils, although some studies also examine their purified forms. Cinnamaldehyde is largely investigated as the principal aldehyde component of cinnamon oil but is frequently tested as an isolated compound under controlled experimental conditions. Terpinen-4-ol is mainly evaluated as the major bioactive constituent of tea tree oil (*M. alternifolia*), while eugenol is typically studied either as the principal phenolic component of clove oil (*S. aromaticum*) or as a purified compound in antifungal assays. These distinctions help clarify how MIC values reported across studies relate either to whole essential oils or to their individual bioactive constituents. Cinnamon (*Cinnamomum* spp.), containing eugenol and cinnamaldehyde as major constituents, exhibited inhibitory activity against *C. albicans*, with minimum inhibitory concentration (MIC) values of approximately 0.125 μg/mL (Tullio et al., 2007; Shahina et al., 2022). Nevertheless, antifungal potency should be interpreted in comparison with conventional antifungal agents tested under similar standardized conditions. For example, fluconazole MICs against susceptible *C. albicans* isolates typically range from 0.125 to 1 μg/mL, whereas amphotericin B MICs generally fall between 0.25 and 1 μg/mL under CLSI broth microdilution assays (Berkow et al., 2020). While some phenolic-rich EOs report MIC values within or near these ranges, many crude oils exhibit higher inhibitory concentrations, indicating that direct equivalence to conventional drugs should be interpreted cautiously and within standardized methodological frameworks (de Aquino Lemos et al., 2009). Likewise, palmarosa oil (*Cymbopogon martinii*), which contained geraniol displayed a 100 percent inhibition of the visible growth after 24 h at the concentration of 0.125–0.25 mg/mL under broth microdilution conditions. This finding however represents inhibition at the endpoint of MIC and does not necessarily imply fungicidal action because MFC determination or time-kill kinetic analysis was not specifically stated (Scalas et al., 2018). True fungicidal action requires confirmation by minimal fungicidal concentration (MFC) determination or time-kill kinetic assays demonstrating ≥99.9% reduction in viable colony-forming units. Moreover, reported MIC values should not be interpreted in isolation, as antifungal susceptibility testing methods exhibit substantial variability between CLSI-standardized broth microdilution assays and non-standard assays, which can dramatically affect the reported potency (Berkow et al., 2020).

*Satureja montana* and *T. vulgaris* essential oils have been reported to exhibit MIC values ranging from 0.01 to 0.25 μg/mL. (Swamy et al., 2016; Tullio et al., 2019), but these very low values are not typical of crude whole essential oils but may be due to the testing of phenolic-enriched fractions or isolated active constituents or to a set of favorable experimental conditions instead of the unrefined oil preparations. Thus, it is necessary to provide a clarification on whether MICs are crude oils, purified compounds or formulated systems when interpreting potency (Bidaud et al., 2021). The heterogeneity in reporting MIC values is highly dependent on the methodological differences which encompasses the choice of solvent systems, inoculum density, incubation time and endpoint determination. DMSO (dimethyl sulfoxide), ethanol or Tween 80 are commonly used solubilizing agents for hydrophobic compounds, namely, thymol, carvacrol, and eugenol. However, the concentration of these solvents may affect membrane permeability and possibly augment or conceal antifungal activity; Cinnamaldehyde, terpinen-4-ol, etc., are also dissolved in DMSO or alcohol-based carriers and variation in the percentage of solvent used in studies may also contribute to variability of reported MICs. Inoculum densities of 10^3^–10^5^ CFU/mL also affect growth kinetics and susceptibility outcomes. Moreover, endpoint determination differs among visual turbidity measurement, spectrophotometric optical density measurement, and metabolic measurement (XTT reduction), with different sensitivities. The visual MIC endpoints can overestimate inhibition, whereas metabolic activity assays can detect residual fungal viability even when growth is suppressed. These methodological discrepancies make it difficult to compare the antifungal potency across laboratories (Berkow et al., 2020; Pezantes-Orellana et al., 2024). Conversely, lemongrass (*Cymbopogon citratus*) oil exhibited higher MIC values (65–250 μg/mL) but demonstrated up to 95% reduction of pre-formed mature biofilm biomass at 5% v/v, as determined by quantitative biomass assays (e.g., crystal violet staining) (Adukwu et al., 2016). This refers specifically to disruption of established biofilms rather than inhibition of initial biofilm formation, a biologically distinct process involving extracellular matrix degradation and increased susceptibility of embedded fungal cells. Such activity is clinically relevant, as mature biofilms are typically more resistant to conventional antifungal therapy. MIC values in this section are mostly related to broth microdilution tests in the CLSI mode especially in cases where individual purified constituents are being tested as thymol, carvacrol, cinnamaldehyde, terpinen-4-ol or eugenol. By contrast, % (v/v) are typically reported to be related to whole crude essential oils or antibiofilm assays, in which the dispersion of oils is done volumetrically, as opposed to the dispersion of them as single compounds. The µg/mL to % (v/v) is not directly proportional due to the variability of compositional variability of essential oils being a multi-component mixture with varying densities, depending on the batch. Normalization based on mass would be determined accurately by determining the density and quantifying the complete compositional content of each of the oil preparations used. Thus, variation in units of reporting is actually indicative of methodological differences but not comparable concentration formats, and one should take care when comparing antifungal potency in different studies.

### 3.2 Activity against other pathogenic and agriculturally relevant fungi

EOs are also active against filamentous and dermatophytic fungi to a significant extent. Oil of oregano, *O. vulgare* EO, in which carvacrol represents the dominant constituent (63.8%), has been reported to inhibit *Fusarium* spp., *C. auris*, and dermatophytes with MIC values of 0.01–0.078 μg/mL. However, such extremely low MIC values are uncommon for crude essential oils and may instead reflect testing of phenolic-enriched fractions, isolated carvacrol, or optimized experimental preparations rather than the unrefined oil itself. In addition, methodological variability between studies, including differences in solvent systems used for oil dispersion, inoculum density, and non-standardized susceptibility testing protocols, can significantly influence reported MIC values. Therefore, these findings should be interpreted cautiously, although they still highlight the strong antifungal contribution of phenolic-rich EO profiles dominated by carvacrol. *C. albicans*, dermatophytes, and *Aspergillus* spp. were highly susceptible to clove (*S. aromaticum*) oil containing 80.9% eugenol, with a large MIC range (0.125–500 ug/mL), mostly through membrane permeabilization and biofilm disruption. Oils of tea tree (*M. alternifolia*) and peppermint (*Mentha piperita*) with 0.12–2.0 ug/mL activity demonstrated the effect of *Candida* spp. and dermatophytes for cell membrane damage, biofilm development, and growth (Merad et al., 2021). Although lavender (*Lavandula angustifolia*) oil had a moderate antifungal effect (MIC = 100–500 ug/mL), it also has an anti-inflammatory effect and is therefore an effective complementary therapy.

### 3.3 Antifungal efficacy measurements

Overall, MIC and MFC values indicate that EOs exhibit variable antifungal activity depending on their chemical composition. Oils with high phenols (especially those containing thymol, carvacrol, and eugenol) tend to exhibit greater inhibitory action than do non-phenolic oils at sub-micromolar concentrated levels, whereas monoterpenes-based oils (e.g., citral, menthol, linalool) have intermediate levels but significant ones (Yu et al., 2022). Table 3 summarizes the data on antifungal activity. Reported inhibitory concentrations below micromolar levels, however, are more likely to result from isolated phenolic constituents or optimized formulations rather than crude EOs and should therefore be viewed with scepticism. Variations in experimental design and reporting units also contribute to differences in antifungal efficacy across the research.

**TABLE 3:** *In vitro* antifungal efficacy of EOs against pathogenic fungi.

Essential oil (active compounds)	Target fungi	MIC (µg/mL)	Mechanism of action	Synergistic partners	References
Cinnamon (*Cinnamomum* spp.) — Eugenol, Cinnamaldehyde	*Candida albicans*, *Aspergillus niger*, *A. flavus*	0.125	Membrane disruption, ergosterol binding	Amphotericin B	Yuan et al. (2024)
Palmarosa (*Cymbopogon martinii*) — Geraniol	*Candida albicans* (stationary phase)	0.125–0.25	Complete eradication within 24 h	-	Inouye et al. (2001)
*Satureja montana* — Carvacrol	*Candida albicans*	0.125–0.25	Fungicidal activity, hyphal inhibition	Amphotericin B (FICI 0.375)	Dalleau et al. (2008)
Thyme (*Thymus vulgaris*) — Thymol, Carvacrol	*Candida auris*, *Aspergillus* spp.*,* Dermatophytes	0.01–0.025	Phenolic membrane disruption	Amphotericin B (additive FICI 0.625)	Jafri and Ahmad (2020)
Oregano (*Origanum vulgare*) — Carvacrol (63.8%)	*Fusarium* spp.*,* Dermatophytes, *Candida auris*	0.01–0.078	Phenolic action, biofilm inhibition	Fluconazole (additive)	Pina-Vaz et al. (2004)
Clove (*Syzygium aromaticum*) — Eugenol (80.9%)	*Candida albicans, Dermatophytes, Aspergillus* spp.	0.125–500	Membrane permeabilization, biofilm disruption	Micafungin/Terbinafine	Braga et al. (2007)
Lemongrass (*Cymbopogon citratus*) — Citral, Geranial	*Candida albicans, Candida auris*	65–250	Biofilm eradication (95% at 5% v/v)	Fluconazole or Amphotericin B	Tyagi et al. (2014)
Tea Tree (*Melaleuca alternifolia*) — Terpinen-4-ol	*Candida* spp.*, Dermatophytes*	0.12–2.0	Membrane disruption	Thyme oil/Oregano oil	Hammer (2002)
Peppermint (*Mentha piperita*) — Menthol, Menthyl acetate	*Candida* spp.*, Alternaria* sp.	0.12–2.0	Biofilm inhibition, fungistatic activity	Fluconazole/Amphotericin B	Rosato et al. (2018)
Lavender (*Lavandula angustifolia*) — Linalool, Linalyl acetate	*Candida albicans, Dermatophytes*	100–500	Anti-inflammatory, moderate antifungal	Fluconazole	D’Auria et al. (2005)

EOs exhibit variable antifungal efficacy depending on their chemical composition and the target organism. Oils rich in phenols from *Cinnamomum*, *Thymus*, *Origanum*, and *Syzygium* species have often been reported to exhibit lower MIC values (Table 3), in a few cases even approaching those of conventional antifungal agents (D’Auria et al., 2005; Kedia et al., 2014; Hou and Huang, 2024). These phenolic constituents have antifungal activity mainly by disrupting membranes, interfering with ergosterol function, inhibiting biofilm formation, and disrupting cellular integrity. In addition, the EOs’ components were shown to exhibit synergistic activity with antifungal agents, such as amphotericin B and fluconazole. Nevertheless, antifungal potency comparisons should be made across studies, considering differences in experimental design, formulations, and reporting standards. The overall findings suggest that EOs, particularly phenolic and terpenoid-rich products, exhibit broad-spectrum antifungal activity. Their usefulness in *Candida* spp. sporozoide and filamentous form of antifungal products emphasize the potential of their use in the form of natural antifungal alternatives or antifungal boosters to the traditional agents (Tullio et al., 2019). They can be used in clinical practice to mitigate growing antifungal resistance, enhance therapeutic efficacy, and reduce toxicity in such oils. They are potentially important as bio-preservatives or crop-protective agents because they effectively prevent *Aspergillus*, *Penicillium*, and *Fusarium* species, are eco-friendly, and do not pollute the environment. Hence, EOs have shown potential as versatile candidates for medical and agricultural antifungal applications.

## 4 Modes of action of antifungal compounds

**FIGURE 1:**
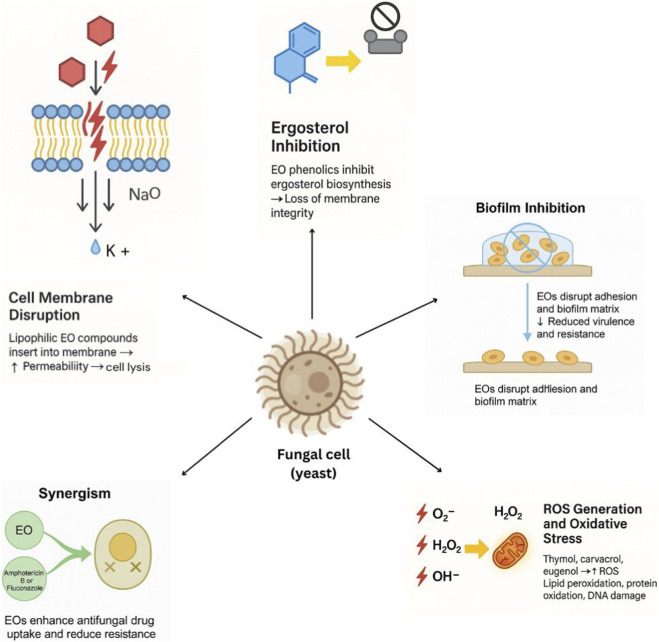
EOs act on multiple targets within the fungal cell.

The mode of antifungal action of EOs (EOs) and their bioactive constituents consists of a series of overlapping, biochemical, and biophysical processes. Insights into these mechanisms facilitate the rational design of EO-based antifungals, combination therapies, and developmental strategies shown in Figure 1. The following are the dominant pathways described in recent literature.

### 4.1 Destabilization of fungal cell membrane and inhibition of ergosterol biosynthesis

Numerous EO compounds are hydrophobic (e.g., terpenoids, phenols, and aldehydes) and integrate into or interact with the fungal lipid bilayers, provoking membrane perturbation (Dehghan-Nayeri et al., 2025). This increases membrane fluidity, disrupts integrity, and releases cytoplasmic contents (ions, ATP, etc.), ultimately leading to cell lysis. Interactions with ergosterol, the principal sterol of the fungal membrane, are reported. For instance, thymol interacts with ergosterol, causing membrane impermeabilization, as with other antifungal drugs (E.g., Amphotericin). Carvacrol and thymol also disrupt ergosterol synthesis. They can block enzymes in that pathway (e.g., squalene epoxidase) (Kowalczyk, 2024). The results in the depletion of ergosterol from the membrane and further damage.

### 4.2 Generation of ROS and oxidative stress

Other mechanisms are associated with the induction of oxidative stress. Some EO components (thymol, carvacrol, eugenol, linalool, etc.) can create overproduction of ROS (superoxide, hydrogen peroxide, and hydroxyl radicals) within the fungal cells. These ROS damage lipids (lipid peroxidation), proteins (enzyme inactivation), and nucleic acids (DNA damage), resulting in cell death.

These oxidative effects, however, may be a consequence of membrane impairment (ion and potential leakage) or via direct metabolic interference. In a few studies, calcium fluxes and vacuolar disturbances were observed as effects of carvacrol-induced stress, which ultimately increased the ROS burden.

### 4.3 Inhibition of strain germination, hyphal/morphological development, and other metabolic pathways

EOs and their constituents can also suppress sporangiospore (spore) germination, an essential step in the infection cycle of pathogenic fungi. For example, EO of *T. vulgaris* and thymol prevented germination of *Rhizopus oryzae* spores.

They can also prevent the yeast-to-filamentous or -hyphal transition (a trait crucial to *Candida* virulence). The investigation focuses on the antifungal activity and mode of action of various EOs (Molaeitabari and Dahms, 2025). Research on thyme, cinnamon, clove, and lemon EOs has shown that they can inhibit the transition of yeast cells into mycelial or hyphal forms (Rajkowska et al., 2019).

Other metabolic pathways are also potentially targeted, including ion homeostasis (e.g., potassium efflux), mitochondrial function, enzyme inhibition (i.e., enzymes in cell wall synthesis or maintenance), as well as telomerase, which is negatively affected (in yeast models).

### 4.4 Anti-biofilm activity and inhibition of adhesion/virulence factors

Biofilms are a particularly serious issue in fungal infections due to their role in conferring drug and immunological resistance to most antifungal drugs. Certain EO compounds have been described as active against biofilm formation or in disturbing mature biofilms (Shariati et al., 2022). For instance, carvacrol has been shown to dramatically reduce *C. albicans* biofilms.

Several EOs have been shown to reduce non-specific adhesion to host surfaces and prosthetic devices, as well as to downregulate virulence factors such as enzyme secretion and morphological switching (Molaeitabari and Dahms, 2025). For example, a study demonstrated that these oils suppress the yeast-to-mycelium transition, a key virulence determinant in *Candida* species (Benzaid et al., 2019).

### 4.5 Synergism among the different EO components

As EOs are composed of various compounds, several bioactive molecules can function in a complementary or synergistic manner (Niu et al., 2020; Ostrowsky et al., 2020; Di Vito et al., 2024). For instance, the combination of thymol and carvacrol often exhibits greater antifungal activity than either compound alone.

By contrast, a mechanistic study in *Saccharomyces cerevisiae* suggested that terpenoid phenols (including carvacrol) induce signalling responses (e.g., calcium bursts, alterations in vacuolar pH), disrupt ion homeostasis, cause metabolic stress, and upregulate stress response genes (Berkow et al., 2020; Bidaud et al., 2021; Tariq et al., 2025). This indicates that their activity is not solely due to direct membrane damage but also involves a cellular saturation response.

## 5 Specific EOs and key active compounds

Specific EOs, their principal active compounds, comparative antifungal potencies, and known mechanisms of action are summarized in Table 4. The strongest antifungal properties are found in the oils of oregano, thyme, and cinnamon, which often have the lowest MICs against *Aspergillus*, *Penicillium*, and *Candida*. These oils primarily affect fungal viability by disrupting membranes, inhibiting ergosterol biosynthesis, and inducing oxidative stress. Carvacrol and thymol disrupt the ion homeostasis and inhibit the spore germination. Tea tree and clove oils offer additional therapeutic benefits, including the reduction of biofilms and topical safety.

**TABLE 4:** Specific EOs and key active compounds.

Essential Oil	Major active compound(s)	Comparative potency/Spectrum	Known mode(s) of action	References
Oregano essential oil (*Origanum* spp.*)*	Carvacrol (often dominant), thymol, p-cymene, γ-terpinene	Very strong activity against *Candida albicans* and non-albicans, *Aspergillus*, *Penicillium*, etc. MICs often low (e.g., in studies comparing EOs against *Aspergillus* spp.*,* oregano had among the lowest MICs)	Disruption of membrane integrity; binding to ergosterol; induction of ROS; inhibits spore germination. Carvacrol causes calcium fluxes and ion homeostasis disruption and resembles TOR-pathway stress in yeast model	Hossain et al. (2016)
Thyme essential oil (*Thymus vulgaris*)	Thymol (major), also carvacrol, p-cymene etc.	Similar potency to oregano in many fungal pathogens. In some grain storage studies, thyme is less efficient than oregano for mycelial inhibition but still effective especially against spores	Acts on ergosterol (interaction and biosynthesis inhibition), membrane disruption, suppression of spore germination; also affects ion efflux	Paster et al. (1995)
Cinnamon (‘cassia’) essential oil (*Cinnamomum* spp.*)*	Cinnamaldehyde, eugenol (in some types), possibly others (cinnamic acid derivatives)	Very potent, often among the top oils in *Candida* studies. In *Investigation …* study, cinnamon oil had MICs as low as 0.002% (v/v) for many clinical isolates	Cinnamaldehyde is known to inhibit cell wall/membrane functions, may interfere with ergosterol biosynthesis; also inhibits hyphal transition/morphological virulence; can affect ion leakageetc.	Gucwa et al. (2018)
Tea Tree oil (*Melaleuca alternifolia*)	Terpinen-4-ol, γ-terpinene, α-terpineol etc.	Moderately strong antifungal against *Candida* and dermatophytes; sometimes less potent than oregano/thyme/cinnamon but valued for broad safety and topical use	Membrane disruption, leakage; reduction in biofilms; sometimes inhibition of virulence factors. Some studies show potentiation in combination. Less data on ergosterol interference compared to oregano/thymol/cinnamon	Hammer (2002)
Clove oil (*Syzygium aromaticum*)	Eugenol is major; also, β-caryophyllene, others	Strong antifungal, especially against *Candida*, dermatophytes; sometimes somewhat slower-acting or needing higher doses than oregano/*Thymus*/cinnamon but has potent activity	Eugenol causes membrane damage, interacts with ergosterol, causes leakage of ions; also contributes to ROS generation. In some studies, clove oil inhibited the yeast-to-mycelial transition and affected metabolic enzymes	Pinto et al. (2009)

### 5.1 Mechanisms underlying antifungal activity

**FIGURE 2:**
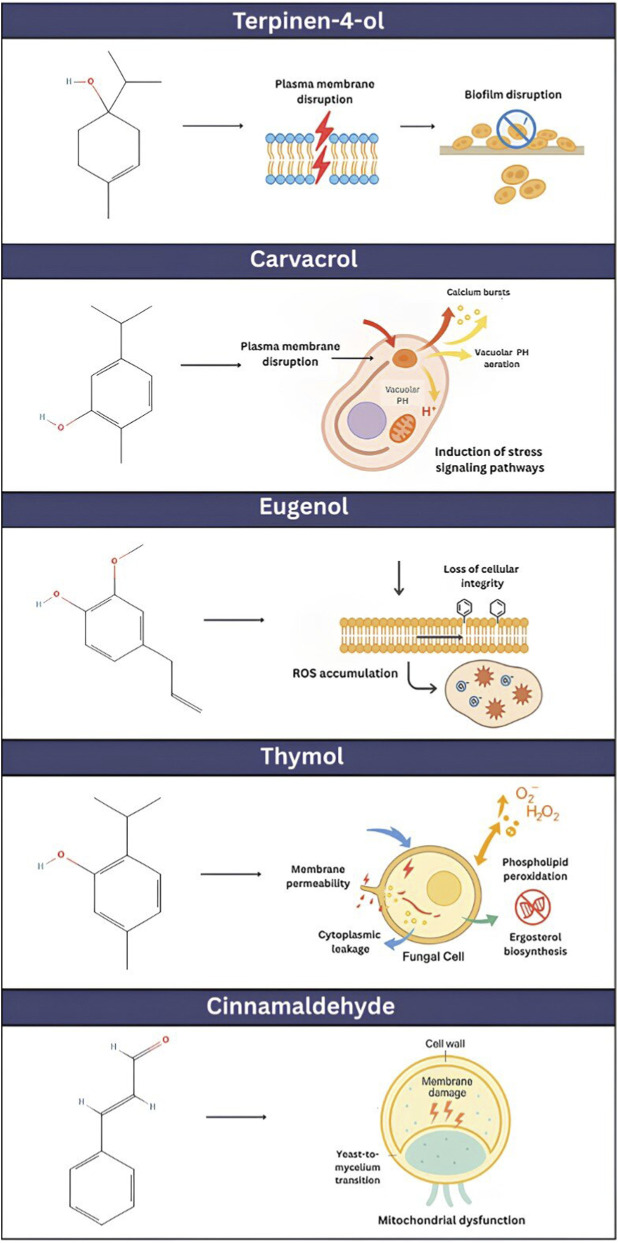
Mechanism of action of major compounds present in EOs as antifungal agents. Chemical structures were retrieved from the PubChem database (NIH, USA) (Kim et al., 2021).

Below are some of the key compounds and what is known about their mechanisms at the molecular level:

- Thymol: It attaches to ergosterol and effects the membrane permeability. It induces cytoplasmic leakage and phospholipid peroxidation with the production of ROS and inhibits genes related to ergosterol (sterol) biosynthesis shown in Figure 2 (Zhang et al., 2019).
- Carvacrol: Another membrane-disrupting compound structurally and functionally related to thymol is carvacrol (also a phenolic monoterpene), which is recognised for its ability to disrupt membranes. Cellular effects, such as calcium bursts and alterations in vacuolar pH, have been described in yeast models. These primary events trigger major stress responses, such as those to autophagy and metabolic reprogramming, to respond to the damage as depicted in Figure 2 (Tariq et al., 2025).
- Cinnamaldehyde: Its major mode of action is against membrane damage. It was proposed that it can affect the integrity of cell wall moieties and the general stability of the cell membrane. In addition to its direct antimicrobial actions, Cinnamaldehyde has been demonstrated to modulate the yeast-to-mycelium transition and hyphal growth in *Candida* species, as shown in Figure 2.
- Eugenol: It affects the cell membrane and induces a loss in cellular integrity. In addition to the physical interference, it triggers oxidative stress in the cell (Figure 2).
- Terpinen-4-ol (derived from tea tree oil): The main component in tea tree oil, predominantly acts as a membrane-damaging agent. This basic action undermines the microbial cell’s defences. On the other hand, it has demonstrated promising antimicrobial activity in practice, with biofilm inhibition illustrated in Figure 2.

## 6 Synergistic effects with conventional antifungals

EOs exhibit synergistic effects when combined with conventional antifungal agents such as amphotericin B, fluconazole, and itraconazole. This combination can enhance antifungal efficacy, reduce required drug doses, and mitigate resistance mechanisms. Table 5 highlights the synergistic interactions between EOs and conventional antifungal drugs, emphasizing their potential to enhance therapeutic efficacy and overcome resistance.

**TABLE 5:** Synergistic effects with conventional antifungals.

Essential oil	Conventional drug	Observed effect	References
Lemongrass (*Cymbopogon citratus*)	Amphotericin B, Itraconazole	Strong inhibitory effect on resistant *Aspergillus* spp. strains; reduced MIC values	Allizond et al. (2023)
Clove (*Syzygium aromaticum*)	Fluconazole	Enhanced permeability and antifungal action against *Candida albicans*	Bravo-Chaucanés et al. (2022)
Cinnamon (*Cinnamomum verum*)	Azoles	Potentiated fungicidal activity; synergistic effects confirmed via FICI ≤0.5	Cavallo et al. (2024)
Tea Tree (*Melaleuca alternifolia*)	Fluconazole	Improved cell permeability, additive to synergistic effect	Kong et al. (2019)
Geranium (*Pelargonium graveolens*)	Amphotericin B	Fungistatic and fungicidal synergy against *Aspergillus fumigatus*	Allizond et al. (2023)

Methods for evaluating synergy include the Checkerboard Microdilution test and Time-Kill assays.

The Fractional Inhibitory Concentration Index (FICI) is calculated as:

FICI = (MIC_EO combination/MIC_EO alone) + (MIC_drug combination/MIC_drug alone).

Interpretation: ≤0.5 = Synergy; 0.5–1 = Additive; 1–2 = Indifferent; ≥2 = Antagonism.

## 7 Applications beyond medicine

Beyond clinical uses, EOs have applications in food preservation, agriculture, and nanotechnology-based delivery systems, summarized in Table 6. Essential or volatile oils, by and large, have a lot of additional uses besides hospitals; they are used in food preservation, agriculture, and some future nanotechnology-powered systems. For example, citrus and herbal EOs such as lemon, orange, coriander, and thyme are extremely effective at killing fungi that cause fruit and vegetable rot, e.g., *Penicillium digitatum* and *Penicillium expansum*, thereby extending product shelf life. Moreover, EOs vapours and sprays can be employed in the agricultural sector to effectively control phytopathogenic fungi, such as *Fusarium* and *Alternaria*, which are the main sources of disease by inhibiting spore germination and reducing pathogen dissemination. Nanotechnology-based innovations have paved the way for major breakthroughs for EOs in that: nanoencapsulation is able to deliver EOs more stably, with a controlled release and a higher antifungal effect, and a nano-emulsion can provide higher solubility and bioavailability even at a lower dosage. By and large, these punctual instances of EOs’ use across various fields exemplify their potential as a major source of biologically based solutions for the planet’s sustainability across different sectors.

**TABLE 6:** Applications of EOs.

Application area	Target organisms/Purpose	Outcome/Mechanism	References
Food Preservation	*Penicillium digitatum, Penicillium expansum*	EOs (lemon, orange, coriander, thyme) inhibit spoilage fungi; extend shelf life	Falleh et al. (2020)
Agriculture	Phytopathogenic fungi (e.g., *Fusarium, Alternaria*)	EO vapors and sprays inhibit spore germination and disease spread	Allizond et al. (2023)
Nanoencapsulation	EOs for food and pharma delivery	Improves stability, controlled release, enhanced antifungal action	Merad et al. (2021)
Nanoemulsion Technology	EO formulations for crops/food systems	Boosts solubility and bioavailability while lowering dosage requirements	Falleh et al. (2020)

## 8 Formulation and delivery approaches

Bioactive plant compounds such as essential oils (EOs) and other natural agents (Jha et al., 2012; 2014; Suganya et al., 2017) possess considerable therapeutic and agricultural potential; however, their practical application is often limited by inherent physicochemical properties, including high volatility, chemical instability, photosensitivity, and poor aqueous solubility. These factors contribute to rapid degradation and inconsistent bioavailability, particularly in systemic delivery. To address these limitations, nanotechnology-based delivery systems and solvent-modified formulations have been explored to enhance dispersibility, stability, and controlled release of EO constituents. Although such nano formulations can improve formulation stability and apparent bioavailability, quantitative pharmacokinetic evaluation remains limited. Key parameters commonly used to assess systemic drug exposure such as maximum plasma concentration (Cmax), area under the concentration–time curve (AUC), and tissue distribution are rarely reported for EO-based nanocarriers. In many studies, claims of improved “bioavailability” are inferred primarily from enhanced solubility, increased dispersion, or improved *in vitro* antifungal activity rather than demonstrated increases in systemic exposure or tissue penetration *in vivo*. Consequently, while nanocarrier-based delivery systems represent a promising strategy for improving EO formulation performance, further *in vivo* pharmacokinetic and efficacy studies are required to determine whether improved solubility and stability translate into meaningful therapeutic outcomes. Allizond et al., revealed considerable antifungal effectiveness of lemongrass, clove, and geranium EOs against drug-resistant *Aspergillus* spp.*,* while also noting that their volatility and hydrophobic nature hinder viable formulation in water-based pharmaceutical systems (Allizond et al., 2023). This highlights the need for encapsulation methods such as nanoemulsions, liposomes, and polymeric nanoparticles to stabilise the oils, decrease evaporation, and ensure prolonged activity (Yammine et al., 2022). Likewise, the review in Molecules on EO-based antifungals for onychomycosis (Alghaith et al., 2021) examined the development of topical nanoemulsion creams and hydrogel matrices capable of targeted local delivery via the nail bed or stratum corneum. These sophisticated formulations can promote local absorption of active ingredients and may reduce irritation compared with traditional ethanol-based or ointment-based formulations. Nevertheless, the arguments of decreased systemic toxicity are largely formulation- and dose-dependent and cannot yet be substantiated with a large-scale *in vivo* safety assessment.

A new use of NADES is among the most significant developments in the science of formulations. It has been reported that NADES are sustainable and biodegradable extraction and solubilization media that can enhance the stability and solubility of poorly-water-soluble phytochemicals to enhance antifungal activity against *C. albicans* (Espino et al., 2019; Miryala et al., 2021). Although these benefits exist, the toxicological assessment of NADES is minimal, especially when taken orally, because safety depends heavily on the constituent chemical dose and the exposure pathway. Furthermore, pharmaceutical preparations based on NADES have yet to be accepted by regulatory authorities, and issues remain in standardization, mass production, and long-term safety testing. It therefore follows that although NADES-sourced systems appear to show great promise in topical and localized antifungal applications, additional toxicological, regulatory, and scalability research is needed before pharmaceutical applications can be widely adopted. Additionally, Pezantes-Orellana et al., noted that incorporating EOs into micro- and nanoencapsulation systems, Pickering emulsions, and hydrogels can significantly enhance bioavailability and shelf life while regulating the release rate of active ingredients (Merad et al., 2021).

Topical treatments are the most effective method for addressing fungal infections on the skin, scalp, and nails, as localised delivery diminishes systemic exposure and reduces adverse side effects. Nevertheless, systemic therapy can be a plausible approach to treating fungal infections, but its feasibility depends strongly on preclinical evidence. To improve stability during circulation and protect EOs constituents against rapid degradation, liposomes, nanoemulsions, and other nanocarriers have been suggested However, most of these studies have been limited to *in vitro* experiments or initial *in vivo* models. Although formulation methods like nanoencapsulation, NADES-based extraction, and controlled-release system have the capability to enhance the stability, dispersibility, and local bioavailability of EO constituents, their ability to lead to systemic therapeutic effects reliably has not been determined yet. This means that these strategies will require a lot of *in vivo* validation, pharmacokinetic characterization and extensive safety profiling before they can be considered viable in clinical or large-scale therapeutic use.

## 9 Safety, toxicological profile, and regulatory considerations

Antifungal effect of EOs and plant-derived constituents are widely reported, but their safety profiles are highly dose-dependent and require systematic quantitative evaluation. Cytotoxic effects have been reported in mammalian cell lines at concentrations approaching antifungal MICs, with half-maximum inhibitory concentrations (IC 50) typically ranging between 100–500 μg mL^-1^ depending on the composition of the oil, exposure time, and cell phenotype. Investigations of acute toxicity in animal models also indicate moderate toxicity of many EO constituents, where oral LD 50 ranges are 1–5 g kg^-1^ of thymol, carvacrol, and eugenol. In addition to systematic toxicity, the irritation thresholds must be considered because phenolic components such as thymol and eugenol have been reported to cause mucosal or skin irritations at higher concentrations depending on the exposure conditions and formulations. These quantitative toxicity metrics are necessary to put antifungal MIC values into perspective and define possible therapeutic ranges, especially when using antifungals in a systemic or high-dose setting. Therefore, careful dose optimization and extensive toxicological assessment is essential to balance the effects of antifungals with tolerable safety levels (Dhifi et al., 2016). In this regard, the selectivity index (SI), which is the ratio of IC50 to MIC, is an important parameter for assessing the feasibility of an antifungal agents, as it approximates the therapeutic window. It has been reported that whereas certain EOs show positive SI values (>10), indicating selective antifungal activity, others exhibit narrow tolerances, with effective MIC or MFC levels close to cytotoxic levels, and thus can cause drug toxicity. The results of the study indicate the need to combine quantitative toxicity parameters, such as IC50, LD50, and irritation thresholds, with antifungal efficacy data to conduct a meaningful risk-benefit analysis and formulation optimization.

The classification of regulatory EO-based products poses serious difficulties for their clinical and commercial translation, because regulatory conditions vary across intended uses and formulations. EO-based products can be controlled depending on their use as botanical drugs, herbal medicinal products, cosmetics, or biopesticides, each having different approval avenues. An example is that therapeutic formulations to treat an illness must undergo rigorous testing as botanical drugs under regulations such as those of the U.S. Food and Drug Administration or as herbal medicinal products under the European Medicines Agency, where quite extensive toxicological, pharmacokinetic, and clinical efficacy data are required.

The Botanical Drug Development pathway and US Food and Drug Administration’s (FDA) are some of the regulatory frameworks that must be followed when developing medicinal formulations incorporating EOs. Before receiving regulatory approval, it requires thorough characterisation of the botanical source material, meticulous preclinical toxicity investigation, pharmacokinetic profiling, and strictly regulated clinical trials. Similar to this, the European Medicines Agency (EMA) may approve EO-containing pharmaceuticals within the EU under the Traditional Herbal Medicinal Products Directive (THMPD), which calls for substantial proof of safety, consistent product quality, and documented traditional medicinal use. A proven track record of therapeutic use, standardised quality standards, and safety data are the requirements for authorisation under this directive (Singh, 2001; Pezantes-Orellana et al., 2024). On the other hand, the EU (European Union) Cosmetic Regulation (EC No. 1223/2009) or similar national safety regulations govern EO-derived compounds used in cosmetic applications. These rules allow substances like linalool, limonene, and eugenol to be utilised as fragrance compounds as long as they meet predetermined safety evaluations and concentration restrictions. Lastly, EO-based antifungal compositions are usually categorised as biopesticides when applied in agricultural contexts. These products are registered under the Environmental Protection Agency’s (EPA) biopesticide program in the United States. Prior to approval, the program assesses environmental safety, acceptable residue levels on crops, and potential ecological implications.

Formulation is also vital in countering the toxicity of essential oils. Experimental findings have demonstrated that encapsulation of essential oils into nano-carrier systems, e.g., nanoliposomes, nanoniosomes, etc., reduces cytotoxic effects relative to free oils and can be used to enhance biocompatibility without loss of biological activity (Emtiazi et al., 2022). In a similar vein, Espino et al. (2019) found that formulations using NADES can lower cytotoxic effects by minimising solvent residue and enhancing the uniformity of dispersion (Espino et al., 2019). However, the authors warn that the safety of the components in NADES also requires toxicological assessment, as their safety has not been universally confirmed.

In addition to human safety, environmental and agricultural regulatory issues must be considered. An article in Phytochemistry Reviews (2025) noted that while EO-based bio-fungicides are biodegradable, they require ecotoxicity evaluations and assessments of non-target effects before deployment on a large agricultural scale (Esmaeili et al., 2025). The absence of standardised guidelines across regions complicates approval processes, as each regulatory body uses varying criteria for efficacy, safety, and environmental sustainability. Thus, systematic safety testing, which should include cytotoxicity analyses, dermal sensitisation evaluations, ecotoxicological studies, and chronic exposure information, is essential to support regulatory approval of EO-based therapeutics and biocontrol agents.

## 10 Limitations and challenges

Despite the research conducted to develop antifungal strategies relying on the use of EOs, the problem of clinical and commercial use of antifungal products remains largely unaddressed (Singh, 2001; Natu and Tatke, 2019). First of all, the most significant issue here is variability in chemical composition, which depends on such factors as the source of the plant, its natural environment, and the method of extraction (Hudaib et al., 2002; Falleh et al., 2020). Because of this variability, standardisation, quality control and reproducibility of results generated by various research groups become an issue (Bizzo et al., 2023).

The most notable barrier is cited as the lack of properly designed clinical research and standardised testing procedures. A majority of the strong evidence is restricted to *in vitro* research, and hence, the results of the attempts can hardly be compared to each other, nor can the efficacy be accurately detected (Konuk and Ergüden, 2020). Furthermore, it is also not well known that microorganisms may develop resistance to sublethal levels of EOs that will cast doubt on the long-term usefulness of antibacterial agents (Bakkali et al., 2008).

The most severe barriers are cytotoxicity and dose optimisation. Although in most instances the antifungal effect of EO is potent, its use is limited by a narrow therapeutic index, which results in safety problems (Allizond et al., 2023). Even though encapsulation and formulation methods have helped to improve the stability and delivery, there is still a big challenge in balancing the potency and safety (Merad et al., 2021). Finally, absence of clear connections between structure and activity, structure and pharmacokinetic data are hindrances to registration and clinical application of the agents (Raut and Karuppayil, 2014). The authors feel that the further research should be guided towards analytical standardisation, biologically proven-assays, sophisticated formulation design, and application of multidisciplinary teams, which will be significant in translation of vital oil-based antifungal agents into clinical practice (Espino et al., 2019).

## 11 Conclusion

EOs offer a diverse range of natural compounds that are both adaptable and environmentally friendly. They exhibit antifungal activity in both medical and agricultural applications by operating through multiple pathways. They disrupt cell membranes, interfere with ergosterol biosynthesis, and generate oxidative stress. They also lower the pathogen viability by acting as broad-spectrum antifungal agents, unlike most conventional antifungal agents. In preclinical trials, combining EOs with antifungal agents such as amphotericin B or fluconazole has been shown to improve antifungal efficacy and reduce resistance development, largely through synergistic interactions that enable lower doses of conventional therapy. However, there is still substantial evidence of lower toxicity in *in vitro* and early *in vivo* models, and the requirements have yet to be confirmed at the clinical stage. Combining the essential oils with activity of agents like amphotericin B or fluconazole has been demonstrated to enhance antifungal activity and counteract the development of resistance in preclinical research, mostly due to the synergistic effects, which might permit a decrease in dosage of conventional medicines, but any observed resulting reduction of toxicity is only supported by preclinical findings. Recent advancements in formulation have significantly improved performance. Methods such as nanoencapsulation and NADES-based systems enhance the solubility of the volatile elements. These techniques further enhance stability and optimise drug delivery. The EOs’ composition can vary greatly by batch, and existing clinical testing is scarce, which is why establishing common safety standards and uniform regulatory policies is necessary. When conducting future studies, particular consideration should be given to the standardization of antimicrobial susceptibility testing systems and standardized MIC and MFC systems to enhance comparability between studies. In addition, selectivity indices (SI = IC50/MIC) must be characterized systematically to determine therapeutic ranges and to inform safe formulation context. It needs to conduct preclinical validation of the drug in relevant models of fungal infection *in vivo* and extensive pharmacokinetic and biodistribution studies to bridge the gap between *in vitro* efficacy and clinical relevance. Finally, properly designed preclinical trials on dosage, safety, and the efficacy of the formulation will be required to transfer the oil-based antifungal strategies to other effective therapeutic interventions. Translational research is a process that connects laboratory discoveries to clinical practice, and thus, the treatments developed are reliable and useful for patients. Therefore, EOs can become a viable alternative to innovative, versatile antifungal agents, expanding opportunities to transform how pests and agriculture in general are treated.

## References

[B1] Adukwu E. C. Bowles M. Edwards-Jones V. Bone H. (2016). Antimicrobial activity, cytotoxicity and chemical analysis of lemongrass essential oil (Cymbopogon flexuosus) and pure citral. Appl. Microbiol. Biotechnol. 100, 9619–9627. 10.1007/s00253-016-7807-y 27562470 PMC5071368

[B2] Ahmad Khan A. Shoaib Amjad M. Saboon (2019). GC-MS analysis and biological activities of *Thymus vulgaris* and *Mentha arvensis* essential oil. Turkish J. Biochem. 44, 388–396. 10.1515/tjb-2018-0258

[B3] Aimad A. Youness E. A. Sanae R. El Moussaoui A. Bourhia M. Salamatullah A. M. (2022). Chemical composition and antifungal, insecticidal and repellent activity of essential oils from Origanum compactum benth. Used in the mediterranean diet. Front. Plant Sci. 13, 798259. 10.3389/fpls.2022.798259 35371154 PMC8964369

[B4] Alghaith A. F. Alshehri S. Alhakamy N. A. Hosny K. M. (2021). Development, optimization and characterization of nanoemulsion loaded with clove oil-naftifine antifungal for the management of tinea. Drug Deliv. 28, 343–356. 10.1080/10717544.2021.1879314 33517791 PMC8725874

[B5] Allizond V. Cavallo L. Roana J. Mandras N. Cuffini A. M. Tullio V. (2023). *In vitro* antifungal activity of selected essential oils against drug-resistant clinical aspergillus spp. strains. Molecules 28, 7259. 10.3390/molecules28217259 37959679 PMC10650698

[B6] Bakkali F. Averbeck S. Averbeck D. Idaomar M. (2008). Biological effects of essential oils – a review. Food Chem. Toxicol. 46, 446–475. 10.1016/j.fct.2007.09.106 17996351

[B7] Basu S. Debroy R. Kumar H. Singh H. Ramaiah S. Anbarasu A. (2023). Bioactive phytocompounds against specific target proteins of *Borrelia recurrentis* responsible for louse‐borne relapsing fever: genomics and structural bioinformatics evidence. Med. Vet. Entomol. 37, 213–218. 10.1111/mve.12623 36377635

[B8] Benzaid C. Belmadani A. Djeribi R. Rouabhia M. (2019). The effects of mentha × piperita essential oil on C. albicans growth, transition, biofilm formation, and the expression of secreted aspartyl proteinases genes. Antibiotics 8, 10. 10.3390/antibiotics8010010 30704020 PMC6466576

[B9] Berkow E. L. Lockhart S. R. Ostrosky-Zeichner L. (2020). Antifungal susceptibility testing: current approaches. Clin. Microbiol. Rev. 33, e00069-19. 10.1128/CMR.00069-19 32349998 PMC7194854

[B10] Bidaud A.-L. Schwarz P. Herbreteau G. Dannaoui E. (2021). Techniques for the assessment of *in vitro* and *in vivo* antifungal combinations. J. Fungi 7, 113. 10.3390/jof7020113 33557026 PMC7913650

[B11] Bizzo H. R. Brilhante N. S. Nolvachai Y. Marriott P. J. (2023). Use and abuse of retention indices in gas chromatography. J. Chromatogr. A 1708, 464376. 10.1016/j.chroma.2023.464376 37717451

[B12] Braga P. C. Sasso M. D. Culici M. Alfieri M. (2007). Eugenol and thymol, alone or in combination, induce morphological alterations in the envelope of Candida albicans. Fitoterapia 78, 396–400. 10.1016/j.fitote.2007.02.022 17590533

[B13] Bravo-Chaucanés C. P. Vargas-Casanova Y. Chitiva-Chitiva L. C. Ceballos-Garzon A. Modesti-Costa G. Parra-Giraldo C. M. (2022). Evaluation of anti-candida potential of Piper nigrum extract in inhibiting growth, yeast-hyphal transition, virulent enzymes, and biofilm formation. J. Fungi 8, 784. 10.3390/jof8080784 36012773 PMC9409899

[B14] Casadevall A. Kontoyiannis D. P. Robert V. (2019). On the emergence of candida auris: climate change, azoles, swamps, and birds. mBio 10, e01397–19. 10.1128/mBio.01397-19 31337723 PMC6650554

[B15] Cavallo L. Menotti F. Roana J. Costa C. Longo F. Pagano C. (2024). Synergistic effect of essential oils and antifungal agents in fighting resistant clinical isolates of Candida auris. Pharmaceutics 16, 957. 10.3390/pharmaceutics16070957 39065654 PMC11279409

[B16] Daferera D. J. Ziogas B. N. Polissiou M. G. (2003). The effectiveness of plant essential oils on the growth of botrytis cinerea, fusarium sp. and Clavibacter michiganensis subsp. michiganensis. Crop Prot. 22, 39–44. 10.1016/S0261-2194(02)00095-9

[B17] Dalleau S. Cateau E. Bergès T. Berjeaud J.-M. Imbert C. (2008). *In vitro* activity of terpenes against candida biofilms. Int. J. Antimicrob. Agents 31, 572–576. 10.1016/j.ijantimicag.2008.01.028 18440786

[B18] de Aquino Lemos J. Rodrigues Costa C. Rodrigues de Araújo C. Kioko Hasimoto Souza L. do Rosário Rodrigues Silva M. (2009). Susceptibility testing of Candida albicans isolated from oropharyngeal mucosa of hiv + patients to fluconazole, amphotericin b and caspofungin. killing kinetics of caspofungin and amphotericin b against fluconazole resistant and susceptible isolates. Braz. J. Microbiol. 40, 163–169. 10.1590/s1517-83822009000100028 24031337 PMC3768489

[B19] de Sousa D. P. Damasceno R. O. S. Amorati R. Elshabrawy H. A. de Castro R. D. Bezerra D. P. (2023). Essential oils: chemistry and pharmacological activities. Biomolecules 13, 1144. 10.3390/biom13071144 37509180 PMC10377445

[B20] Dehghan-Nayeri D. Asgarpanah J. Shams-Ghahfarokhi M. Seyedjavadi S. S. Saremi G. Eslamifar A. (2025). Antifungal activity, mechanistic insights, and combinatorial effects of Pycnocycla bashagardiana essential oil against Aspergillus fumigatus. South Afr. J. Bot. 181, 272–280. 10.1016/j.sajb.2025.04.015

[B21] Dhifi W. Bellili S. Jazi S. Bahloul N. Mnif W. (2016). Essential oils’ chemical characterization and investigation of some biological activities: a critical review. Medicines 3, 25. 10.3390/medicines3040025 28930135 PMC5456241

[B22] Di Vito M. Rosato R. Rizzo S. Cacaci M. Urbani A. Sanguinettii M. (2024). Enhancing fluconazole reactivation against *Candida auris*: efficacy of *Cinnamomum zeylanicum* essential oil *versus* cinnamaldehyde. Microbiol. Spectr. 12, e0017624. 10.1128/spectrum.00176-24 38483141 PMC10986320

[B23] Diánez F. Santos M. Parra C. Navarro M. J. Blanco R. Gea F. J. (2018). Screening of antifungal activity of 12 essential oils against eight pathogenic fungi of vegetables and mushroom. Lett. Appl. Microbiol. 67, 400–410. 10.1111/lam.13053 30022505

[B24] D’Auria F. D. Tecca M. Strippoli V. Salvatore G. Battinelli L. Mazzanti G. (2005). Antifungal activity of *Lavandula angustifolia* essential oil against *Candida albicans* yeast and mycelial form. Med. Mycol. 43, 391–396. 10.1080/13693780400004810 16178366

[B25] Emtiazi H. Salari Sharif A. Hemati M. Fatemeh Haghiralsadat B. Pardakhti A. (2022). Comparative study of nano‐liposome and nano‐niosome for delivery of *Achillea millefolium* essential oils: development, optimization, characterization and their cytotoxicity effects on cancer cell lines and antibacterial activity. Chem. Biodivers. 19, e202200397. 10.1002/cbdv.202200397 36097678

[B26] Esmaeili A. Saleh I. Abu-Dieyeh M. H. (2025). Antifungal potential of plant-based extracts against candida species: values, safety concerns, and possible applications. Phytochem. Rev. 24, 5801–5844. 10.1007/s11101-025-10093-x

[B27] Espino M. Solari M. Fernández M. de los Á. Boiteux J. Gómez M. R. Silva M. F. (2019). NADES-mediated folk plant extracts as novel antifungal agents against Candida albicans. J. Pharm. Biomed. Anal. 167, 15–20. 10.1016/j.jpba.2019.01.026 30738239

[B28] Fahed L. Khoury M. Stien D. Ouaini N. Eparvier V. El Beyrouthy M. (2017). Essential oils composition and antimicrobial activity of six conifers harvested in Lebanon. Chem. Biodivers. 14. 10.1002/cbdv.201600235 27685246

[B29] Falleh H. Ben Jemaa M. Saada M. Ksouri R. (2020). Essential oils: a promising eco-friendly food preservative. Food Chem. 330, 127268. 10.1016/j.foodchem.2020.127268 32540519

[B30] Gao T. Zhou H. Zhou W. Hu L. Chen J. Shi Z. (2016). The fungicidal activity of thymol against Fusarium graminearum *via* inducing lipid peroxidation and disrupting ergosterol biosynthesis. Molecules 21, 770. 10.3390/molecules21060770 27322238 PMC6272974

[B31] Geremia N. Brugnaro P. Solinas M. Scarparo C. Panese S. (2023). Candida auris as an emergent public health problem: a current update on European outbreaks and cases. Healthcare 11, 425. 10.3390/healthcare11030425 36767000 PMC9914380

[B32] Ghosh S. Basu S. Anbarasu A. Ramaiah S. (2025a). A comprehensive review of antimicrobial agents against clinically important bacterial pathogens: prospects for phytochemicals. Phytotherapy Res. 39, 138–161. 10.1002/ptr.8365 39496516

[B33] Ghosh S. Basu S. Kayal T. Ashok G. Ramaiah S. Anbarasu A. (2025b). Computational advancements to facilitate therapeutic application of phytochemicals: where do we stand? Discov. Appl. Sci. 7, 491. 10.1007/s42452-025-06772-1

[B34] Gucwa K. Milewski S. Dymerski T. Szweda P. (2018). Investigation of the antifungal activity and mode of action of Thymus vulgaris, citrus limonum, Pelargonium graveolens, cinnamomum cassia, Ocimum basilicum, and Eugenia caryophyllus essential oils. Molecules 23, 1116. 10.3390/molecules23051116 29738503 PMC6099571

[B35] Gupta A. K. Venkataraman M. (2022). Antifungal resistance in superficial mycoses. J. Dermatological Treat. 33, 1888–1895. 10.1080/09546634.2021.1942421 34132155

[B36] Hammer K. A. Carson C. F. Riley T. V. (2002). *In vitro* activity of Melaleuca alternifolia (tea tree) oil against dermatophytes and other filamentous fungi. J. Antimicrob. Chemother. 50, 195–199. 10.1093/jac/dkf112 12161399

[B37] Hossain F. Follett P. Dang Vu K. Harich M. Salmieri S. Lacroix M. (2016). Evidence for synergistic activity of plant-derived essential oils against fungal pathogens of food. Food Microbiol. 53, 24–30. 10.1016/j.fm.2015.08.006 26678126

[B38] Hou G. Huang T. (2024). Essential oils as promising treatments for treating Candida albicans infections: research progress, mechanisms, and clinical applications. Front. Pharmacol. 15, 1400105. 10.3389/fphar.2024.1400105 38831882 PMC11145275

[B39] Hudaib M. Speroni E. Di Pietra A. M. Cavrini V. (2002). GC/MS evaluation of thyme (Thymus vulgaris L.) oil composition and variations during the vegetative cycle. J. Pharm. Biomed. Anal. 29, 691–700. 10.1016/S0731-7085(02)00119-X 12093498

[B40] Inouye S. Takizawa T. Yamaguchi H. (2001). Antibacterial activity of essential oils and their major constituents against respiratory tract pathogens by gaseous contact. J. Antimicrob. Chemother. 47, 565–573. 10.1093/jac/47.5.565 11328766

[B41] Jafri H. Ahmad I. (2020). Thymus vulgaris essential oil and thymol inhibit biofilms and interact synergistically with antifungal drugs against drug resistant strains of Candida albicans and Candida tropicalis. J. Mycol. Med. 30, 100911. 10.1016/j.mycmed.2019.100911 32008964

[B42] Jha D. K. Panda L. Lavanya P. Ramaiah S. Anbarasu A. (2012). Detection and confirmation of alkaloids in leaves of Justicia adhatoda and bioinformatics approach to elicit its anti-tuberculosis activity. Appl. Biochem. Biotechnol. 168, 980–990. 10.1007/s12010-012-9834-1 22899014

[B43] Jha D. K. Panda L. Ramaiah S. Anbarasu A. (2014). Evaluation and comparison of radical scavenging properties of solvent extracts from Justicia adhatoda leaf using DPPH assay. Appl. Biochem. Biotechnol. 174, 2413–2425. 10.1007/s12010-014-1164-z 25185502

[B44] Jiménez-Reyes M. F. Carrasco H. Olea A. F. Silva-Moreno E. (2019). Natural compounds: a sustainable alternative to the phytopathogens control. J. Chil. Chem. Soc. 64, 4459–4465. 10.4067/S0717-97072019000204459

[B45] Joshi T. Sharma T. R. Panickar A. Mathpal S. Guchhait R. Ramaiah S. (2025). Screening of natural compounds potentially inhibiting the mutated C-terminal domain of GyrA in Salmonella typhi using ML-based *in silico* approach. Netw. Model. Analysis Health Inf. Bioinforma. 14, 129. 10.1007/s13721-025-00632-z

[B46] Kedia A. Prakash B. Mishra P. K. Dubey N. K. (2014). Antifungal and antiaflatoxigenic properties of Cuminum cyminum (L.) seed essential oil and its efficacy as a preservative in stored commodities. Int. J. Food Microbiol. 168–169, 1–7. 10.1016/j.ijfoodmicro.2013.10.008 24211773

[B47] Khaleel C. Tabanca N. Buchbauer G. (2018). α-Terpineol, a natural monoterpene: a review of its biological properties. Open Chem. 16, 349–361. 10.1515/chem-2018-0040

[B48] Khoury M. Stien D. Eparvier V. Ouaini N. El Beyrouthy M. (2016). Report on the medicinal use of eleven lamiaceae species in Lebanon and rationalization of their antimicrobial potential by examination of the chemical composition and antimicrobial activity of their essential oils. Evidence-Based Complementary Altern. Med. 2016, 2547169. 10.1155/2016/2547169 28053641 PMC5178328

[B92] Kim S. Chen J. ChengKim S. Chen J. Cheng T. Gindulyte A. (2021). PubChem in 2021: new data content and improved web interfaces. Nucleic Acids Res. 49, D1388–D1395. 10.1093/nar/gkaa971 33151290 PMC7778930

[B49] Köhler J. R. Hube B. Puccia R. Casadevall A. Perfect J. R. (2017). Fungi that infect humans. Microbiol. Spectr. 5. 10.1128/microbiolspec.FUNK-0014-2016 28597822 PMC11687496

[B50] Kong Q. Zhang L. An P. Qi J. Yu X. Lu J. (2019). Antifungal mechanisms of α‐terpineol and terpene‐4‐alcohol as the critical components of *Melaleuca alternifolia* oil in the inhibition of rot disease caused by *Aspergillus ochraceus* in postharvest grapes. J. Appl. Microbiol. 126, 1161–1174. 10.1111/jam.14193 30614164

[B51] Konuk H. B. Ergüden B. (2020). Phenolic –OH group is crucial for the antifungal activity of terpenoids *via* disruption of cell membrane integrity. Folia Microbiol. (Praha). 65, 775–783. 10.1007/s12223-020-00787-4 32193708

[B52] Kowalczyk A. (2024). Essential oils against candida auris—A promising approach for antifungal activity. Antibiotics 13, 568. 10.3390/antibiotics13060568 38927234 PMC11200742

[B53] Lyman M. Forsberg K. Sexton D. J. Chow N. A. Lockhart S. R. Jackson B. R. (2023). Worsening spread of *Candida auris* in the United States, 2019 to 2021. Ann. Intern. Med. 176, 489–495. 10.7326/M22-3469 36940442 PMC11307313

[B54] Marriott P. J. Shellie R. Cornwell C. (2001). Gas chromatographic technologies for the analysis of essential oils. J. Chromatogr. A 936, 1–22. 10.1016/S0021-9673(01)01314-0 11760992

[B55] Martins G. A. Bicas J. L. (2024). Antifungal activity of essential oils of tea tree, oregano, thyme, and cinnamon, and their components. Braz. J. Food Technol. 27, e2023071. 10.1590/1981-6723.07123

[B56] Mazumder I. Rehman M. Deshmukh F. Shah S. Singh A. Ramaiah S. (2026). A review on novel strategies to combat multidrug resistance in pathogenic bacteria exploiting synergism between essential oil and antibiotics. World J. Microbiol. Biotechnol. 42, 36. 10.1007/s11274-025-04775-z 41504837

[B57] Merad Y. Derrar H. Belmokhtar Z. Belkacemi M. (2021). Aspergillus genus and its various human superficial and cutaneous features. Pathogens 10, 643. 10.3390/pathogens10060643 34071092 PMC8224566

[B58] Miryala S. K. Basu S. Naha A. Debroy R. Ramaiah S. Anbarasu A. (2021). Identification of bioactive natural compounds as efficient inhibitors against *Mycobacterium tuberculosis* protein-targets: a molecular docking and molecular dynamics simulation study. J. Mol. Liq. 341, 117340. 10.1016/j.molliq.2021.117340

[B59] Miryala S. K. Basu S. Naha A. Debroy R. Ramaiah S. Anbarasu A. (2022). Datasets comprising the quality validations of simulated protein-ligand complexes and SYBYL docking scores of bioactive natural compounds as inhibitors of *Mycobacterium tuberculosis* protein-targets. Data Brief. 42, 108146. 10.1016/j.dib.2022.108146 35479419 PMC9035630

[B60] Molaeitabari A. Dahms T. E. S. (2025). Blocking the shikimate pathway amplifies the impact of carvacrol on biofilm formation in *Candida albicans* . Microbiol. Spectr. 13, e0275424. 10.1128/spectrum.02754-24 39918333 PMC11878086

[B61] Natu K. N. Tatke P. A. (2019). Essential oils – prospective candidates for antifungal treatment? J. Essent. Oil Res. 31, 347–360. 10.1080/10412905.2019.1604437

[B62] Niu C. Wang C. Yang Y. Chen R. Zhang J. Chen H. (2020). Carvacrol induces Candida albicans apoptosis associated with Ca2+/Calcineurin pathway. Front. Cell. Infect. Microbiol. 10, 192. 10.3389/fcimb.2020.00192 32426298 PMC7203418

[B63] Noble B. A. Jurcic Smith K. L. Jones J. D. Galvin B. W. Timbrook T. T. (2023). *Candida auris* rates in blood culture on the rise: results of US surveillance. Microbiol. Spectr. 11, e0221623. 10.1128/spectrum.02216-23 37623375 PMC10580899

[B64] Ostrowsky B. Greenko J. Adams E. Quinn M. O’Brien B. Chaturvedi V. (2020). *Candida auris* isolates resistant to three classes of antifungal medications — new York, 2019. MMWR Morb. Mortal. Wkly. Rep. 69, 6–9. 10.15585/mmwr.mm6901a2 31917780 PMC6973342

[B65] Paster N. Menasherov M. Ravid U. Juven B. (1995). Antifungal activity of oregano and thyme essential oils applied as fumigants against fungi attacking stored grain. J. Food Prot. 58, 81–90. 10.4315/0362-028X-58.1.81 31121777

[B66] Pérez-Cantero A. López-Fernández L. Guarro J. Capilla J. (2020). Azole resistance mechanisms in aspergillus: update and recent advances. Int. J. Antimicrob. Agents 55, 105807. 10.1016/j.ijantimicag.2019.09.011 31542320

[B67] Pezantes-Orellana C. German Bermúdez F. Matías De la Cruz C. Montalvo J. L. Orellana-Manzano A. (2024). Essential oils: a systematic review on revolutionizing health, nutrition, and omics for optimal well-being. Front. Med. (Lausanne). 11, 1337785. 10.3389/fmed.2024.1337785 38435393 PMC10905622

[B68] Pina‐Vaz C. Gonçalves Rodrigues A. Pinto E. Costa‐de‐Oliveira S. Tavares C. Salgueiro L. (2004). Antifungal activity of *thymus* oils and their major compounds. J. Eur. Acad. Dermatology Venereol. 18, 73–78. 10.1111/j.1468-3083.2004.00886.x 14678536

[B69] Pinto E. Vale-Silva L. Cavaleiro C. Salgueiro L. (2009). Antifungal activity of the clove essential oil from Syzygium aromaticum on candida, aspergillus and dermatophyte species. J. Med. Microbiol. 58, 1454–1462. 10.1099/jmm.0.010538-0 19589904

[B70] Rahman A. (2014). Antifungal potential of essential oil and ethanol extracts of *Lonicera japonica* thunb. against dermatophytes. EXCLI J. 13, 427–436. 10.17877/DE290R-15589 26417269 PMC4464489

[B71] Rajkowska K. Nowicka-Krawczyk P. Kunicka-Styczyńska A. (2019). Effect of clove and thyme essential oils on candida biofilm Formation and the oil distribution in yeast cells. Molecules 24, 1954. 10.3390/molecules24101954 31117281 PMC6572016

[B72] Rao A. Zhang Y. Muend S. Rao R. (2010). Mechanism of antifungal activity of terpenoid phenols resembles calcium stress and inhibition of the TOR pathway. Antimicrob. Agents Chemother. 54, 5062–5069. 10.1128/AAC.01050-10 20921304 PMC2981246

[B73] Raut J. S. Karuppayil S. M. (2014). A status review on the medicinal properties of essential oils. Ind. Crops Prod. 62, 250–264. 10.1016/j.indcrop.2014.05.055

[B74] Rosato A. Carocci A. Catalano A. Clodoveo M. L. Franchini C. Corbo F. (2018). Elucidation of the synergistic action of Mentha piperita essential oil with common antimicrobials. PLoS One 13, e0200902. 10.1371/journal.pone.0200902 30067803 PMC6070247

[B75] Scalas D. Mandras N. Roana J. Tardugno R. Cuffini A. M. Ghisetti V. (2018). Use of Pinus sylvestris L. (Pinaceae), Origanum vulgare L. (Lamiaceae), and Thymus vulgaris L. (Lamiaceae) essential oils and their main components to enhance itraconazole activity against azole susceptible/not-susceptible Cryptococcus neoformans strains. BMC Complement. Altern. Med. 18, 143. 10.1186/s12906-018-2219-4 29724221 PMC5934896

[B76] Shahina Z. Molaeitabari A. Sultana T. Dahms T. E. S. (2022). Cinnamon leaf and clove essential oils are potent inhibitors of Candida albicans virulence traits. Microorganisms 10, 1989. 10.3390/microorganisms10101989 36296264 PMC9607542

[B77] Shariati A. Didehdar M. Razavi S. Heidary M. Soroush F. Chegini Z. (2022). Natural compounds: a hopeful promise as an antibiofilm agent against candida species. Front. Pharmacol. 13, 917787. 10.3389/fphar.2022.917787 35899117 PMC9309813

[B78] Sharifi-Rad J. Sureda A. Tenore G. Daglia M. Sharifi-Rad M. Valussi M. (2017). Biological activities of essential oils: from plant chemoecology to traditional healing systems. Molecules 22, 70. 10.3390/molecules22010070 28045446 PMC6155610

[B79] Singh N. (2001). Trends in the epidemiology of opportunistic fungal infections: predisposing factors and the impact of antimicrobial use practices. Clin. Infect. Dis. 33, 1692–1696. 10.1086/323895 11641825

[B80] Souza E. J. D. de Kringel D. H. Lima Costa I. H. de Hackbart H. C. dos S. Cantillano R. F. F. (2024). Antifungal potential of essential oils from different botanical sources against *Penicillium digitatum*: chemical composition and antifungal mechanisms of action by direct contact and volatile. Nat. Prod. Res. 39, 1–9. 10.1080/14786419.2024.2405865 39295586

[B81] Suganya S. Nandagopal B. Anbarasu A. (2017). Natural inhibitors of HMG‐CoA reductase—an *insilico* approach through molecular docking and simulation studies. J. Cell. Biochem. 118, 52–57. 10.1002/jcb.25608 27216569

[B82] Swamy M. K. Akhtar M. S. Sinniah U. R. (2016). Antimicrobial properties of plant essential oils against human pathogens and their mode of action: an updated review. Evidence-Based Complementary Altern. Med. 2016, 3012462. 10.1155/2016/3012462 28090211 PMC5206475

[B83] Tariq H. Zou W. Wang L. Lv J. Khan A. R. Bilal M. S. (2025). Biofumigation with carvacrol disrupts membrane integrity and metabolic homeostasis in yam leaf spot pathogen *Alternaria alternata* . Pestic. Biochem. Physiol. 213, 106544. 10.1016/j.pestbp.2025.106544 40744588

[B84] Thompson G. R. Young J.-A. H. (2021). Aspergillus infections. N. Engl. J. Med. 385, 1496–1509. 10.1056/NEJMra2027424 34644473

[B85] Tullio V. Nostro A. Mandras N. Dugo P. Banche G. Cannatelli M. A. (2007). Antifungal activity of essential oils against filamentous fungi determined by broth microdilution and vapour contact methods. J. Appl. Microbiol. 102, 1544–1550. 10.1111/j.1365-2672.2006.03191.x 17578419

[B86] Tullio V. Roana J. Scalas D. Mandras N. (2019). Evaluation of the antifungal activity of mentha x piperita (Lamiaceae) of Pancalieri (Turin, Italy) essential oil and its synergistic interaction with azoles. Molecules 24, 3148. 10.3390/molecules24173148 31470602 PMC6749244

[B87] Tyagi A. K. Gottardi D. Malik A. Guerzoni M. E. (2014). Chemical composition, *in vitro* anti-yeast activity and fruit juice preservation potential of lemon grass oil. LWT - Food Sci. Technol. 57, 731–737. 10.1016/j.lwt.2014.02.004

[B88] Yammine J. Chihib N.-E. Gharsallaoui A. Dumas E. Ismail A. Karam L. (2022). Essential oils and their active components applied as: free, encapsulated and in hurdle technology to fight microbial contaminations. A review. Heliyon 8, e12472. 10.1016/j.heliyon.2022.e12472 36590515 PMC9798198

[B89] Yu B. Li C. Gu L. Zhang L. Wang Q. Zhang Y. (2022). Eugenol protects against Aspergillus fumigatus keratitis by inhibiting inflammatory response and reducing fungal load. Eur. J. Pharmacol. 924, 174955. 10.1016/j.ejphar.2022.174955 35436473

[B90] Yuan X. Cao D. Xiang Y. Jiang X. Liu J. Bi K. (2024). Antifungal activity of essential oils and their potential synergistic effect with amphotericin B. Sci. Rep. 14, 31125. 10.1038/s41598-024-82380-0 39732745 PMC11682416

[B91] Zhang J. Ma S. Du S. Chen S. Sun H. (2019). Antifungal activity of thymol and carvacrol against postharvest pathogens botrytis cinerea. J. Food Sci. Technol. 56, 2611–2620. 10.1007/s13197-019-03747-0 31168143 PMC6525678

